# Associations with short-term and long-term use of inhaled corticosteroid in adults with asthma. A retrospective analysis

**DOI:** 10.1590/1806-9282.20241240

**Published:** 2025-07-07

**Authors:** Ariele Pedroso, Joice Mara de Oliveira, Thainá Bessa Alves, Vitória Cavalheiro Puzzi, Natielly Soares Correia, Heloisa Galdino Gumieiro Ribeiro, Marcos Ribeiro, Karina Couto Furlanetto

**Affiliations:** 1 Universidade Pitágoras Unopar Anhanguera, Research and Postgraduate Center – Londrina (PR), Brazil.; 2 Universidade Estadual de Londrina, Laboratory of Research in Respiratory Physiotherapy – Londrina (PR), Brazil.; 3 Universidade Estadual de Londrina, Department of Medicine – Londrina (PR), Brazil.

**Keywords:** Corticosteroids, Asthma, Physical functional, Pulmonary function tests, Exercise tolerance

## Abstract

**OBJECTIVE:**

The objective of this study was to compare anthropometric data and pulmonary and extrapulmonary physical functional outcomes of short- and long-term use of inhaled corticosteroids in adults with asthma.

**METHODS:**

This cross-sectional study with retrospective analysis included clinically stable adults with asthma. Anthropometric data, exacerbation history, pulmonary function, disease control, dyspnea in daily life, quality of life, anxiety, depression, functional exercise capacity, performance in daily activities, body composition, physical activity level, respiratory muscle strength, lower limb strength, and handgrip strength were assessed and compared. Participants were separated into two groups based on the current duration of inhaled corticosteroids use below (short-term) or above (long-term) 3 years (≤3 years [n=37] and >3 years [n=30]) for comparative analysis.

**RESULTS:**

A total of 67 participants were analyzed (69% female, 49±14 years, body mass index 29±6 kg/m², FEV_1_ 2.21±0.79 L). The group ≤3 years presented statistically higher spirometric values and exercise capacity compared to the group >3 years even after analysis of covariance adjustments for age, body mass index, disease control, and treatment duration (forced vital capacity 3.39 [3.09–3.70] L vs. 2.93 [2.59–3.26] L, p=0.048; FEV_1_ 2.40 [2.17–2.63] L vs. 2.00 [1.75–2.25] L, p=0.023; 6-min walk test 567 [536–599] vs. 519 [484–554] m, respectively, p=0.048). All the other variables were similar between groups (p>0.05 for all).

**CONCLUSION:**

Participants who used inhaled corticosteroids for more than 3 years exhibited worse pulmonary function and exercise capacity. Despite this, exacerbation history, disease control, quality of life, anxiety, depression, and dyspnea in daily life, among other physical functional outcomes, were similar between short- and long-term use of inhaled corticosteroids in adults with asthma.

## INTRODUCTION

Asthma is a chronic respiratory disease characterized by chronic inflammation of the airways and by the history of several clinical symptoms that vary in intensity and time. To improve the inflammatory process and control symptoms, individuals with asthma benefit from drug treatment associated with non-pharmacological strategies^1^.

Inhaled corticosteroids (ICS) are the primary anti-inflammatory treatment for asthma, blocking the arachidonic acid cascade^2^. Studies show that ICS help control symptoms, reduce exacerbations and hospitalizations, decrease airway hyperresponsiveness and inflammation, improve quality of life, and lower asthma-related mortality. However, the effects of ICS duration on physical functional outcomes in asthma remain unclear^3^.

The use of the minimum effective ICS dose is recommended, and regular monitoring should be done in consultations for asthma treatment. Some studies argue that high doses of ICS increase the chances of adverse effects, from mild symptoms such as oral candidiasis to severe symptoms such as hypothalamic-pituitary-adrenal (HPA) axis depression^4^. Although adverse effects are present in the use of high doses of ICS, this medication significantly improved the risk-benefit ratio for the treatment of asthma^5^.

Given the uncertainties about physical functional outcomes associated with the prolonged use of ICS in adults with asthma^6^, this study aims to perform a retrospective cross-sectional analysis to compare anthropometric data, pulmonary physical functional (e.g., exacerbation history, pulmonary function, respiratory muscle strength, disease control, and dyspnea in daily life); and extrapulmonary physical functional outcome (e.g., quality of life, anxiety, depression, functionality, performance in daily activities, body composition, physical activity level, exercise capacity, and lower limb strength, and handgrip strength) of adults with asthma on short- or long-term use of ICS.

## METHODS

Patients diagnosed with asthma treated by the outpatient service of the Pulmonology Department at the University Hospital and primary healthcare units in the city of Londrina, Brazil, were invited to participate in the study. Recruitment was conducted through flyers to attract patients with varying degrees of disease severity. This is a cross-sectional study with retrospective analysis of data collected in three consecutive visits (days 1, 4, and 12) between April 2018 and June 2020, in which the validity, reliability, and feasibility of four functional tests were investigated in adults with asthma, following COSMIM recommendations^7^.

The protocol was approved by the Ethics Committee of Unopar University (3.060.314), where the assessments were carried out, and all participants signed the Informed Consent Form. Inclusion criteria were diagnosis of asthma by a pulmonologist according to the Global Initiative for Asthma (GINA)^1^; aged 18 years or more; under medical treatment for at least 6 months; with clinical stability (i.e., no exacerbation or increase in asthma medication for at least 30 days); and the absence of limiting cardiovascular and/or musculoskeletal disease. Exclusion criteria were inability to perform the tests, diagnosis of any other pulmonary disease, and not reporting the duration or dose of the current ICS.

On the first day (visit 1), participants underwent anthropometric measurements and self-reported exacerbation history, which included the use of oral corticosteroids for at least 3 days, an increased need for reliever medications, or hospitalizations/emergency visits due to worsening asthma symptoms. Pulmonary function was assessed using the MicroLab 3500 Spirometer (Care Fusion^®^, Ireland), where reference values corresponded to the Brazilian population^8^. They also completed the Asthma Control Test (ACT), in which 25 points mean total control, ≥20 points for controlled asthma, 16–19 for partially controlled asthma, and ≤15 for uncontrolled asthma^9^. Asthma Quality of Life Questionnaire (AQLQ), which consists of 32 questions where the higher the score, the better the quality of life^10^. Anxiety and depression were assessed using the Hospital Anxiety and Depression Scale (HADS), in which the higher the score, the more symptoms of anxiety and depression^11^. Participants were asked to classify their dyspnea in daily life using the modified Medical Research Council (mMRC) scale; the higher, the greater the dyspnea impact^12^. Additionally, functional tests were performed, including the Timed Up and Go (TUG), the 1-min sit-to-stand test (STS), and the 4-m gait speed (4GMS), all at maximum speed^7^.

After 3 days (visit 2), participants underwent the second set of functional tests. Assessment of performance in activities of daily living (ADL) using the Glittre-ADL test^13^ and the London Chest Activity of Daily Living (LCADL) scale^14^. Measurement of body composition using the Biodynamics 310TM (Biodynamics Corp, Charlottesville, United States)^15^. Participants were also given the Actigraph^®^ wGT3X-BT activity monitor (Pensacola, United States) to objectively measure physical activity (PA), worn at the waist for 7 days, with valid data considered for at least 4 days with a minimum of 480 min per day^16^.

Finally, 8 days later (visit 3), participants underwent two 6-min walk tests (6MWT) in a 30-meter corridor, following the ERS/ATS guidelines with reference values calculated^17^. Measurements of respiratory muscle strength by the maximum expiratory pressure (MEP) and inspiratory (MIP) were conducted using the Manovacuometer (MVD 300 manovacuometer; Homed, São Paulo, Brazil)^18^. Quadriceps and hamstring strength were measured in the preferred lower limb using a tension meter (EMG System^®^, Brazil) during maximal voluntary isometric contraction^19^. Handgrip strength was measured using a hydraulic dynamometer (Jamar^®^) with the participant seated, shoulders in a neutral position, and elbow of the dominant limb at 90°^20^. Muscle strength tests were performed three times each, with the best result selected for analysis. Validated tests and questionnaires were employed for patients with asthma^7–20^.

The duration of ICS use was established through patient self-report. The dose of ICS was calculated as equivalent to budesonide, which was the most used medication, to standardize the values for comparison and analysis. The participants were categorized into two groups based on the median duration of ICS use, coincident with a previous study that used the same measure for group allocation^5^. Those using ICS for 3 years or less were in the short-term group (≤3 years), and those with more than 3 years were in the long-term group (>3 years).

Numerical data normality was assessed using the Shapiro-Wilk test and presented as mean±standard deviation or median [interquartile range 25–75%]. Categorical variables were compared using the chi-square test, while numerical variables were analyzed with Student's t-test and Mann-Whitney U test. Analysis of covariance (ANCOVA) was used to adjust for confounding factors, including age, BMI, disease control, and treatment duration. Statistical significance was p<0.05.

## RESULTS

Of the 250 individuals diagnosed with asthma eligible for the study, 83 (33%) did not meet the inclusion criteria and 80 (32%) refused to participate. A total of 87 participants were initially included, but 20 were excluded for the following reasons: ICS duration not reported (n=7), inability to perform the tests (n=5), exacerbation during the evaluation period (n=5), presence of another pulmonary disease (n=2), and asthma medication change within the last 30 days (n=1). Ultimately, 67 participants were analyzed.

Table 1 shows that most participants (69%) were overweight young adult women. Few had asthma exacerbations in the past year, with 14% requiring hospitalization. Those patients in the group >3 years had reduced pulmonary function regarding FVC and FEV_1_. Moderate and severe asthma were present in 42 and 39% of participants, respectively. The ACT questionnaire showed mostly controlled asthma, and dyspnea was reported only during intense activities on the mMRC scale.

**Table 1:** Comparison of general characteristics and pulmonary physical function outcomes of participants using short- or long-term inhaled corticosteroids.

Variables		Total (n=67)	≤3 years (n=37)	>3 years (n=30)	p
Sex, f/m (%)		46 (69)/21 (31)	26 (70)/11 (30)	20 (67)/10 (33)	0.752
Age, years		49±14	47±13	52±14	0.163
Weight, kg		75±16	75±13	75±19	0.734
Height, cm		161±9	163±9	159±9	0.056
Medication and disease history					
	ICS dose, mcg	800 [381–800]	800 [363–800]	800 [350–800]	0.551
	ICS time, months	36 [12–72]	24 [7–36]	90 [60–120]	**<0.0001**
	LABA dose, mcg	24 [12–24]	24 [9–24]	24 [14–24]	0.188
	LABA time, months	36 [6–72]	12 [5–36]	72 [48–120]	**<0.0001**
	Diagnosis time, months	300 [129–555]	264 [36–468]	456 [153–609]	**0.031**
	Treatment time, months	126 [45–360]	60 [36–236]	168 [81–375]	**0.013**
	Exacerbation, times	3 [2–3]	2 [2–3]	3 [2–5]	0.052
	Hospitalization, times	2 [1–4]	2 [1–3]	3 [2–5]	0.556
Spirometry and respiratory muscle strength					
	FVC, L	3.09 [2.36–3.85]	3.37 [2.45–4.01]	2.81 [2.27–3.47]	**0.046**
	FVC, % pred	88 [78–96]	91 [82–99]	83 [64–92]	**0.047**
	FEV_1_, L	2.29 [1.47–2.85]	2.63 [1.69–2.98]	1.99 [1.27–2.48]	**0.01**4
	FEV_1_, % pred	77 [61–90]	83 [64–92]	69 [53–80]	**0.00**9
	FEV_1_/FVC, %	70 [62–80]	75 [64–80]	69 [54–78]	0.116
	MIP, cmH_2_O#	94±37	97±35	90±37	0.502
	MEP, cmH_2_O#	105±45	109±48	100±40	0.478
Severity and disease control					
	Severity, I/II/III/IV/V, n	1/5/7/28/26	1/3/6/18/9	0/2/1/10/17	0.064
	ACT, points	20 [16–22]	21 [17–23]	19 [15–22]	0.184
Dyspnea impact					
	mMRC, points	1 [1–2]	1 [1–2]	1 [0–2]	0.264

f: female; m: male; ICS: inhaled corticosteroid; LABA: long-acting B2 adrenergic agonists; FVC: forced vital capacity; FEV_1_: forced expiratory volume in the first second; MIP: maximal inspiratory pressure; MEP: maximal expiratory pressure; I: intermittent; II: mild persistent; III: moderate persistent; IV: severe persistent; V: severe asthma; ACT: Asthma Control Test; mMRC: modified Medical Research Council Scale. Data are described in absolute value, mean±SD or median [interquartile range 25–75%]. Statistically significant values are indicated in bold.

# Variables with predicted values were also calculated and compared (P>0.05 for all).

Table 2 shows no differences between groups in functional exercise capacity, performance, and self-reported limitations in daily activities. Questionnaire results (quality of life, anxiety, and depression), body composition, peripheral muscle strength, physical activity, and sedentary behavior were also similar.

**Table 2:** Comparison of extrapulmonary physical functional outcomes of participants using short- or long-term inhaled corticosteroids.

Variables		Total (n=67)	≤3 years (n=37)	>3 years (n=30)	p
Functional exercise capacity					
	6MWT, meters#	536 [486–596]	568 [488–610]	528 [480–574]	0.095
	TUG, s#	7.50 [6.34–8.34]	6.42 [5.94–7.24]	6.89 [6.48–7.45]	0.121
	STS 1 min, repetitions#	26 [22–29]	27 [21–32]	24 [22–28]	0.591
	4MGS, m/s#	1.33 [1.14–1.48]	1.33 [1.19–1.54]	1.30 [1.12–1.42]	0.189
Performance and self-reported limitation in activity of daily living					
	Glittre—ADL, min#	3.38 [3.00–3.96]	3.40 [2.98–4.01]	3.29 [3.03–3.94]	0.959
	LCADL, points	21±7	23±7	20±6	0.294
Questionnaires (quality of life, anxiety, and depression)					
	AQLQ, points	5 [4–6]	5 [4–6]	5 [4–6]	0.495
	HADS anxiety, points	8 [4–10]	8 [2–10]	8 [4–12]	0.360
	HADS depression, points	7 [2–9]	5 [2–9]	7 [3–10]	0.354
Body composition					
	Fat-free mass, %	67 [62–74]	68 [62–76]	66 [62–71]	0.525
	Fat mass, %	32 [25–37]	31 [23–37]	32 [28–37]	0.692
	BMI, kg/m²	28±5	28±5	29±6	0.468
Peripheral muscle strength					
	MVIC quadriceps, kgF#	21±12	21±2	21±2	0.895
	MVIC hamstrings, kgF#	12±6	12±1	13±1	0.443
	Handgrip, kgF#	31±11	31±2	31±2	0.926
Physical activity and sedentary behavior					
	Sedentary, h	8.43 [7.38–9.35]	8.18 [7.38–9.35]	8.48 [7.35–9.25]	0.738
	MVPA, h	0.28 [0.13–0.63]	0.38 [0.15–0.73]	0.28 [0.13–0.40]	0.270
	Steps per day, n	5,800 [4,390–9,235]	7,149 [4,655–9,734]	5,574 [4,246–8,049]	0.293

6MWT: 6-min walking test; TUG: timed up and go test; STS: sit to stand test; 4MGS: meter gait speed test; ADL: activity daily living; LCADL: London chest activity of daily living; AQLQ: Asthma Quality of Life Questionnaire; HADS: Hospital Anxiety and Depression Scale; BMI: body mass index; MVIC: maximal voluntary isometric contraction; MVPA: moderate vigorous physical activity.

# Variables with predicted values were also calculated and compared (p>0.05 for all).

In Table 3, after ANCOVA analysis, statistically significant differences were observed for FVC, FEV_1_, and 6MWT. These variables presented worse results in the group with >3 years of ICS; all other variables continued to show no significant differences.

**Table 3:** Comparison of pulmonary function and exercise capacity between participants using short- or long-term inhaled corticosteroids adjusted by analysis of covariance.

Variables	≤3 years (n=37)	>3 years (n=30)	p
FVC, liters	3.39 [3.09–3.70]	2.93 [2.59–3.26]	**0.048**
FEV_1_, liters	2.40 [2.17–2.63]	2.00 [1.75–2.25]	**0.023**
6MWT, meters	567 [536–599]	519 [484–554]	**0.048**

FVC: forced vital capacity; FEV_1_: forced expiratory volume in the first second; 6MWT: 6-min walking test. Data are expressed in weighted means and 95% confidence intervals [95%CI]. The covariates appearing in the model are evaluated at the following values: age (49.26 years), BMI (28.86 kg/m²), ACT score (18.75 points), and treatment duration (194.00 months). Statistically significant values are indicated in bold.

## DISCUSSION

In this study, there were no significant differences between the groups in most of the outcomes assessed. However, adjustments for confounding factors indicated that individuals with longer treatment duration showed lower values in terms of spirometry (FVC and FEV1) and exercise capacity (6MWT), suggesting that, despite prolonged ICS use, there may be a functional decline associated with the natural progression of asthma.

Previous studies indicate that while ICS airway inflammation improves short-term control of asthma, their long-term effect on pulmonary function is less conclusive^21^. In a cohort study, patients on long-term ICS treatment showed slower rates of FEV_1_ decline compared to those not using ICS, but the progression of airway remodeling still contributed to a decline in pulmonary function. This supports the hypothesis that, despite inflammation control, the natural progression of the disease may lead to functional decline even with continuous ICS use^22^.

Indeed, ICS use is a solid therapy among patients with asthma, and positive factors are associated with health^6^. In the present study, both groups were clinically stable and continuously using ICS. We hypothesized that natural impairments in physical functional outcomes due to the progression of the disease might have been attenuated by the ICS use. Moreover, diagnosis time, which was higher in the group >3 years, might have contributed to the differences between groups.

This study does not aim to discourage the use of ICS, which play a fundamental role in controlling the disease^23^. The literature has shown that the disease itself can result in pulmonary functional decline. For example, in a population study, three FEV_1_ measurements were taken over 15 years in 17,506 individuals, including 1,095 with asthma. Individuals with asthma showed a decline in FEV_1_ of 38 mL per year compared to 22 mL per year in normal individuals^24^.

Nonpharmacological strategies, such as promoting physical activity, have shown positive impacts on the health of patients with chronic pulmonary diseases. In addition, pulmonary rehabilitation and the promotion of physical activity are effective and safe for preventing declines in pulmonary function and exercise capacity in patients with chronic pulmonary diseases over time^25^.

These findings emphasize the importance of integrated interventions that combine pharmacological treatments with non-pharmacological strategies to improve functional outcomes in people with asthma. Most physical functional outcomes showed no differences between the groups. These results are considered positive, as prolonged ICS use helped prevent impairments in manageable factors^21^.

This study addresses a gap in the literature by exploring the long-term impact of ICS on physical functional outcomes such as dyspnea, functional capacity, PA, and ADL, with objective, valid, and reliable assessment methods. The main limitations of the study include its single-center, cross-sectional design, retrospective analysis, use of a convenience sample originating from the primary study, and the limited ability to establish causal relationships. Additionally, factors such as medication adherence and the use of oral corticosteroids were not assessed, which may affect the external validity.

## CONCLUSION

Adults with asthma using ICS for more than 3 years presented worse pulmonary function and exercise capacity compared to those on short-term use. However, no significant differences were observed between short- and long-term regarding exacerbation history, disease control, quality of life, anxiety, depression, dyspnea in daily life, or other pulmonary and extrapulmonary physical functional outcomes.

## Data Availability

The datasets generated and/or analyzed during the current study are available from the corresponding author upon reasonable request.
